# Assessment and Validation of Syndromic Case Definitions for Respiratory Syncytial Virus Testing in a Low Resource Population

**DOI:** 10.1097/INF.0000000000002159

**Published:** 2018-08-02

**Authors:** Saad B. Omer, Robert Bednarczyk, Momin Kazi, L. Beryl Guterman, Fatima Aziz, Kristen E. Allen, Inci Yildirim, S. Asad Ali

**Affiliations:** From the * Hubert Department of Global Health; † Department of Epidemiology; ‡ Department of Pediatrics; § Emory Vaccine Center, Emory University, Atlanta, Georgia; ¶ Department of Paediatrics and Child Health, Aga Khan University, Karachi, Pakistan.

**Keywords:** RSV, syndromic case definition, surveillance

## Abstract

Standardized case definitions are needed in decision-making regarding respiratory syncytial virus control strategies, including vaccine evaluation. A syndromic case definition comprising of “wheeze or apnea or cyanosis” could be useful for community-based surveillance of moderate respiratory syncytial virus infection among young infants particularly in resource-limited settings. However, this definition showed modest specificity (29.2%–49.6%), indicating that community-based surveillance may need augmentation with other data.

## Abstract 2

Supplemental Digital Content is available in the text.

Respiratory syncitial virus (RSV) is a major cause of acute lower respiratory infections, particularly among young infants. Vaccine candidates are being developed for maternal immunization to protect infants against RSV.^1^ Decision-making regarding RSV vaccination strategies will require community-based surveillance in a variety of populations. Robust surveillance systems require laboratory testing, which is not available in low-resource settings, and, more importantly, a credible case definition.^2^ For RSV, one has yet to be developed. Previous attempts have relied on retrospective data and/or focused on facility-based cases.^3–5^

This study evaluated potential syndromic case definitions for moderate RSV among young infants in a prospective community-based cohort in Karachi, Pakistan. Case definitions were evaluated using RSV polymerase chain reaction (PCR).

## MATERIALS AND METHODS

### Surveillance

This study was nested in a prospective cohort.^6^ Surveillance was conducted in four low-income settlements in Karachi, Pakistan, which are part of a Demographic Surveillance System. Between February 21, 2015, and April 12, 2016, infants were enrolled (≤10 weeks of age) and followed up through 18 weeks of age with routine evaluation for respiratory syndromes.^6^

Nasopharyngeal swabs were obtained from infants meeting the syndromic screening definition, and total nucleic acid extraction was performed. DNA extracts were tested by PCR for presence of RSV.^3,6,7^

### Screening and Sample Selection

We developed 3 syndromic screening criteria (designed for maximum sensitivity) to identify samples for RSV testing.^6^ For all infants, we compared these screening definitions to first occurrence of symptoms recorded through 12 weeks of age, as the incidence of severe disease was too low between 12 and 18 weeks to be informative. The nasopharyngeal swab associated with this initial visit was tested.

The screening definitions were chosen as they are close approximations of existing surveillance definitions for acute respiratory illnesses. The syndromic screening definitions included: “wheeze or apnea or cyanosis” (the classic manifestation of RSV); “severe chest indrawing with either cough or tachypnea” [severe acute lower respiratory infection (ALRI)]; and “fever or any integrated management of childhood illness (IMCI) danger sign (lethargy or poor feeding confirmed by poor suck or seizure)” (severe pneumonia). Infants could be included in more than one case definition. Our control group consists of infants who only had cough or coryza. Because all nasopharyngeal swabs were obtained from children who met the original syndromic screening definition (Figure S1, http://links.lww.com/INF/D240), no samples were obtained from asymptomatic infants. Therefore, we assess a syndromic case definition for moderate RSV.

### Data Analysis

RSV PCR positivity was compared across each of the 3 case definitions and for control infants. For each case definition, we conducted 2 comparisons:

1. infants who met the given definition compared with infants who did not meet any of the 3 protocol-defined case definitions but had a nasopharyngeal swab obtained after presenting either cough or coryza only
2. infants who met the given definition compared with infants who did not meet the given definition (including infants who met another case definition).

For each comparison, we computed the sensitivity, specificity, positive predictive value (PPV) and negative predictive value (NPV) and plotted the resulting receiver operating characteristic curve. We assessed mean and median age at which the infant met the case definition, stratified by RSV positivity. Additionally, we compared the distribution of symptoms among RSV-positive infants to the RSV-negative infants using a 2-sample *t* test.

Because RSV illness can be more severe in preterm infants, we compared gestational age at birth (categorized ≤31 weeks, 32–36 weeks and ≥37 weeks) against case definition status and RSV positivity using χ^2^ testing. Analyses were conducted using SAS v9.4 (The SAS Institute, Cary, NC), with α = 0.05.

### Ethical Review

The study was approved by Institutional Review Boards at Emory University and the Aga Khan University.

## RESULTS

Of 299 infants included in this study, 30 (12.2%) were RSV PCR positive, 245 (81.9%) met at least one of the protocol-defined case definitions and 54 (18.1%) infants did not meet any of the 3 protocol-defined case definitions but did have a nasopharyngeal swab obtained after presenting either cough or coryza only. Sixty-two (20.7%) infants met more than one case definition concurrently (Table S1 and Figure S2, http://links.lww.com/INF/D240).

**FIGURE 1.**
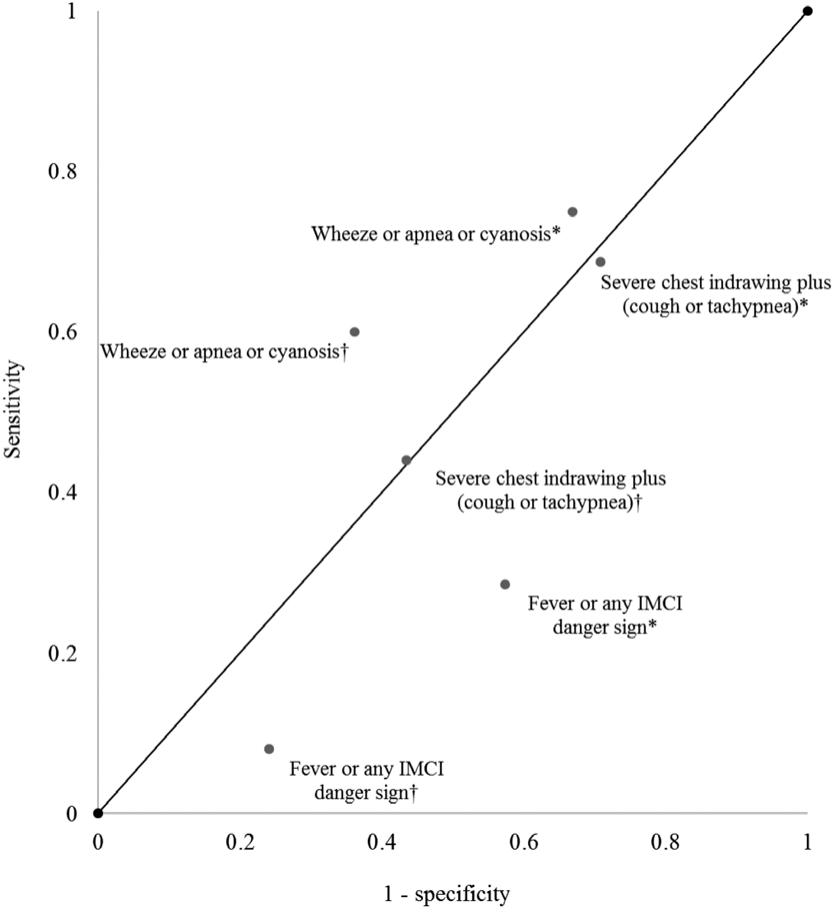
Receiver operating characteristic plot for 3 proposed case definitions for RSV testing among infants 12 weeks of age and younger in urban areas of Karachi Pakistan, with 2 comparison groups per case definition; definitions marked with an asterisk (*) indicate comparison to control infants (ie, infants whose only symptoms were cough or coryza), and definitions marked with a dagger (†) indicate comparison to all infants who did not meet the case definition. The diagonal line represents the line of no discrimination.

Overall, the case definition of “wheeze or apnea or cyanosis” performed the best. In comparison with infants who did not meet any of the 3 protocol-defined case definitions but did have a nasopharyngeal swab obtained after presenting with either cough or coryza only, “wheeze or apnea or cyanosis” had moderate sensitivity and specificity (sensitivity 75%; specificity 33%; PPV 13%; and NPV 91%). For the same comparison, the case definition “severe chest indrawing plus (cough or tachypnea)” demonstrated modest sensitivity and low specificity (sensitivity 68.8%; specificity 29.2%), and the case definition “fever or any IMCI danger signs” demonstrated low sensitivity and specificity (sensitivity 28.6%; specificity 48.6%; Table S2a, http://links.lww.com/INF/D240; Fig. 1).

Compared with all infants who did not meet the case definition (including infants who met another case definition), “wheeze or apnea or cyanosis” also demonstrated moderate sensitivity and specificity (sensitivity 60%; specificity 63.9%; PPV 13%; and NPV 95%). For the same comparison, the case definition “severe chest indrawing plus (cough or tachypnea)” demonstrated modest sensitivity and specificity (sensitivity 44%; specificity 56.6%), and the case definition “fever or any IMCI danger signs” demonstrated low sensitivity and specificity (sensitivity 8%; specificity 75.9%; Table S2b, http://links.lww.com/INF/D240; Fig. 1). Additionally, infants who met the case definition of “fever or any IMCI danger signs” were younger than those who met the other case definitions (Table S3, http://links.lww.com/INF/D240).

Compared with RSV PCR-negative infants, RSV PCR-positive infants were more likely to have exhibited cough (96% versus 79%; *P* = 0.0007) and wheeze (52% versus 32%; *P* = 0.0459) and less likely to have experienced a seizure (0% versus 4%; *P* = 0.0005; Table S3, http://links.lww.com/INF/D240).

Gestational age at birth was available for 259 infants (86.6%); 183 (70.7%) were born at 37 weeks and later, and 10 (3.9%) were born before 32 weeks’ gestation. There were no significant associations between gestational age and meeting any of the case definitions (*P* > 0.05). Similarly, there was no association between gestational age and RSV PCR positivity (χ^2^ *P* value = 0.8655). Additionally, there were no significant associations between gestational age and RSV PCR positivity, when stratified by meeting/not meeting each case definition (data available on request).

## DISCUSSION

Our findings suggest that a syndromic case definition comprising of “wheeze or apnea or cyanosis” could be useful for community-based surveillance of moderate RSV cases among young infants. In our study, this case definition had the highest sensitivity, NPV and PPV compared with the other case definitions across gestational age at birth. However, this definition showed modest specificity indicating that community-based surveillance may need to be augmented with other data. The most specific case definition was “fever or any IMCI danger sign.” We also found that presence of cough and wheeze were independent predictors of laboratory-confirmed RSV infection.

There have been several attempts to optimize case definitions to detect RSV infection and better understand its epidemiology and burden in resource-limited settings. Nyawanda et al^5^ reported that of the standard respiratory case definitions, severe acute respiratory infection (SARI) (acute respiratory infection onset within the last 10 days with fever, cough and hospitalization) had the highest sensitivity (83%; 95% confidence interval: 79–86) with a specificity of 23% (95% confidence interval: 21–24) and suggested using this case definition particularly if implemented in a sentinel influenza surveillance system. However, their analysis was restricted to inpatients, and more sensitive case definitions may be required for evaluating future RSV vaccines in resource-poor settings where RSV testing is not routinely performed. Using case definitions like influenza like illness (ILI) (SARI without hospitalization) and SARI, which are commonly used for influenza surveillance, can underestimate the disease burden by 50%–85% due to lower sensitivities.^8^

Our results corroborate previous findings by Durani et al^9^ who also identified that cough, wheezing and retractions performed the best both as independent predictors for RSV infection in infants and young children and as a combined syndromic case definition with an area under the receiver operating curve (ROC) curve of 0.66 (95% confidence interval: 0.56–0.74). However, their study enrolled facility-based cases up to 36 months of age at the discretion of physicians, while our study included community-based cases up to 12 weeks of age with testing based on syndromic screening definitions. Fever was a common finding among those with and without RSV infection in both studies and was not a good predictor of RSV infection.

There are limited comparable high-quality burden data on RSV among young children from low- and lower–middle-income countries. Most RSV burden studies rely on the surveillance systems for influenza (not optimized for detection of RSV) or retrospective data gathered from medical records. A unique aspect and strength of our study includes the comparison of 3 case definitions with prospective data collection in a well-defined population using a highly sensitive molecular testing for RSV. Moreover, we accounted for gestational age at birth, which is usually not collected in influenza-associated burden surveillance studies.

## Supplementary Material
