# Supplementary material for: Assessment and Validation of Syndromic Case Definitions for Respiratory Syncytial Virus Testing in a Low Resource Population
Source: Pediatr Infect Dis J. 2018 Aug 2;38(3):e57–9. doi: 10.1097/INF.0000000000002159 (PMC6437080; doi:10.1097/INF.0000000000002159)

Contents

Table S1a. Distribution of infants 12 weeks and younger residing in urban areas of Karachi Pakistan meeting one of three potential RSV testing case definitions, and related RSV positivity by case definition. 2

Table S1b. Distribution of infants 12 weeks and younger residing in urban areas of Karachi Pakistan meeting any combination of three potential RSV testing case definitions, and related RSV positivity by case definition. 3

Table S2a. Sensitivity, specificity, and positive and negative predictive values (PPV and NPV, respectively) for three proposed case definitions for RSV testing, with comparison against control infants (i.e. those who did not meet any of the three case definitions). 4

Table S2b. Sensitivity, specificity, and positive and negative predictive values (PPV and NPV, respectively) for three proposed case definitions for RSV testing, with comparison against all other infants who did not meet the specified case definition. 4

Table S3. Mean and median age at diagnosis for infants 12 weeks of age and younger in urban areas of Karachi Pakistan who met one of three potential RSV testing case definitions, stratified by case definition met and RSV PCR test results. 5

Table S4. Comparison of the symptoms presented by RSV positive and RSV negative infants 12 weeks of age and younger in urban areas of Karachi Pakistan. 7

Figure S1. Original syndromic screening case definition. 8

Figure S2: Distribution of infants 12 weeks and younger residing in urban areas of Karachi Pakistan meeting any combination of three potential RSV testing case definitions. Where the size of each bubble is proportional to number of infants meeting each combination of case definitions and in parentheses is the percent of total infants (including those that met none of the protocol-defined case definitions, 54(18.1%)). 9

Table S1a. Distribution of infants 12 weeks and younger residing in urban areas of Karachi Pakistan meeting one of three potential RSV testing case definitions, and related RSV positivity by case definition.

	**Infants meeting case definition**		**Infants with positive RSV test**	
Case definition symptoms	N	%*	N	% (of infants meeting definition)
Wheeze or apnea or cyanosis	114	38.1	15	13.2
Severe chest indrawing plus (cough or tachypnea)	130	43.5	11	8.5
Fever or any IMCI danger sign	68	22.7	2	2.9
Did not meet any of these case definitions	54	18.1	5	9.3

* Percent does not sum to 100 as infants could meet more than one case definition.

Table S1b. Distribution of infants 12 weeks and younger residing in urban areas of Karachi Pakistan meeting any combination of three potential RSV testing case definitions, and related RSV positivity by case definition.

**Case definition symptoms**			**Infants meeting case definition**		**Infants with positive RSV test**	
Wheeze or apnea or cyanosis*	Severe chest indrawing plus (cough or tachypnea) †	Fever or any IMCI danger sign‡	N	%	N	% (of infants meeting definition)
Yes	Yes	Yes	5	1.7	0	0.0
Yes	Yes	No	48	16.1	8	16.7
Yes	No	Yes	5	1.7	5	0.0
Yes	No	No	56	18.7	7	12.5
No	Yes	Yes	4	1.3	0	0.0
No	Yes	No	73	24.4	3	4.1
No	No	Yes	54	18.1	2	3.7
No	No	No	54	18.1	5	9.3

* Total infants meeting this definition = 114 (38.1%)

† Total infants meeting this definition = 130 (43.5%)

‡ Total infants meeting this definition = 68 (22.7%)

Table S2a. Sensitivity, specificity, and positive and negative predictive values (PPV and NPV, respectively) for three proposed case definitions for RSV testing, with comparison against control infants (i.e. those who did not meet any of the three case definitions).

	**Sensitivity (95% CI)**	**Specificity (95% CI)**	**PPV (95% CI)**	**NPV (95% CI)**
Wheeze or apnea or cyanosis	75.0 (50.9, 91.3)	33.1 (25.6, 41.3)	13.2 (7.6, 20.8)	90.7 (79.7, 96.9)
Severe chest indrawing plus (cough or tachypnea)	68.8 (41.3, 89.0)	29.2 (22.4, 36.7)	8.5 (4.3, 14.6)	90.7 (79.7, 96.9)
Fever or any IMCI danger sign	28.6 (3.7, 71.0)	42.6 (33.4, 52.2)	2.9 (0.4, 10.2)	90.7 (79.7, 96.9)

Table S2b. Sensitivity, specificity, and positive and negative predictive values (PPV and NPV, respectively) for three proposed case definitions for RSV testing, with comparison against all other infants who did not meet the specified case definition.

	**Sensitivity (95% CI)**	**Specificity (95% CI)**	**PPV (95% CI)**	**NPV (95% CI)**
Wheeze or apnea or cyanosis†	60.0 (38.7, 78.9)	63.9 (57.9, 69.6)	13.2 (7.6, 20.8)	94.6 (90.3, 97.4)
Severe chest indrawing plus (cough or tachypnea) †	44.0 (24.4, 65.1)	56.6 (50.5, 62.5)	8.5 (4.3, 14.6)	91.7 (86.5, 95.4)
Fever or any IMCI danger sign†	8.0 (1.0, 26.0)	75.9 (70.4, 80.9)	2.9 (0.4, 10.2)	90.0 (85.4, 93.6)

Table S3. Mean and median age at diagnosis for infants 12 weeks of age and younger in urban areas of Karachi Pakistan who met one of three potential RSV testing case definitions, stratified by case definition met and RSV PCR test results.

			Age (weeks)			
**Wheeze or apnea or cyanosis**	RSV PCR result	N Obs	Mean	Std Dev	Median	IQR
Met case definition	Positive	15	6.7	3.3	6	3, 9
	Negative	99	6.9	3.6	7	4, 10
Did not meet case definition, but met other case definition	Positive	10	6.6	4.1	7.5	3, 10
	Negative	175	6	3.7	6	4, 9
**Severe chest indrawing plus (cough or tachypnea)**	RSV PCR result	N Obs	Mean	Std Dev	Median	IQR
Met case definition	Positive	11	6.1	2.9	6	4, 8
	Negative	119	6.7	3.3	7	4, 9
Did not meet case definition, but met other case definition	Positive	14	7.1	4.1	9	3, 10
	Negative	155	6	3.9	6	3, 9
**Fever or any IMCI danger sign**	RSV PCR result	N Obs	Mean	Std Dev	Median	IQR
Met case definition	Positive	2	5.5	6.4	5.5	1, 10

	Negative	66	5.1	3.9	5	2, 8
Did not meet case definition, but met other case definition	Positive	23	6.7	3.4	7	3, 10
	Negative	208	6.7	3.5	7	4, 10

**Controls**	RSV PCR result	N Obs	Mean	Std Dev	Median	IQR
Did not meet any case definition (control)	Positive	5	7.2	4.9	10	3, 10
	Negative	49	6.4	3.4	6	4, 9

Table S4. Comparison of the symptoms presented by RSV positive and RSV negative infants 12 weeks of age and younger in urban areas of Karachi Pakistan.

	**RSV Positive (N = 25)**		**RSV Negative (N = 274)**		
Symptom	N	%	N	%	t-test p-value
Cough	24	96.0%	216	78.8%	0.0007
Wheeze	13	52.0%	88	32.1%	0.0459
Tachypnea	11	44.0%	127	46.4%	0.8222
Severe chest indrawing	11	44.0%	120	43.8%	0.9966
Upper respiratory symptoms, NOS	9	36.0%	152	55.5%	
Coryza	8	32.0%	108	39.4%	0.468
Close exposure to family member with prolonged afebrile cough illness	4	16.0%	43	15.7%	0.9862
Atypical paroxysms only	4	16.0%	15	5.5%	
Post-tussive emesis	3	12.0%	78	28.5%	0.0766
Fever	2	8.0%	52	19.0%	0.0774
Whoop	2	8.0%	15	5.5%	0.6031
Poor feeding confirmed by poor suck	1	4.0%	11	4.0%	0.9943
Cyanosis	1	4.0%	10	3.6%	0.9349
Apnea	1	4.0%	7	2.6%	0.6694
Lethargy/movement only when stimulated	1	4.0%	5	1.8%	0.5986
Seizure	0	0.0%	12	4.4%	0.0005
Mechanical complications	0	0.0%	3	1.1%	0.0833
Conjuctivial injection	0	0.0%	1	0.4%	0.3182

Figure S1. Original syndromic screening case definition.

**Infants presenting with any of the following:**
○ Cough, lasting at least one day,<br>○ Coryza,<br>○ Whoop,<br>○ Apnea,<br>○ Post-tussive emesis,<br>○ Cyanosis,<br>○ Seizure,<br>○ Tachypnea<br>▪ >50 breaths/minute for infants older than 2 months or<br>▪ >60 breaths/minute for infants <2 months,<br>○ Severe chest indrawing,<br>○ Movement only when stimulated (or an alternative definition of lethargy),<br>○ Poor feeding confirmed by poor suck,<br>○ Close exposure to any family member with a prolonged afebrile cough illness, or<br>○ Temperature greater than or equal to 38°C

Figure S2: Distribution of infants 12 weeks and younger residing in urban areas of Karachi Pakistan meeting any combination of three potential RSV testing case definitions. Where the size of each bubble is proportional to number of infants meeting each combination of case definitions and in parentheses is the percent of total infants (including those that met none of the protocol-defined case definitions, 54(18.1%)).

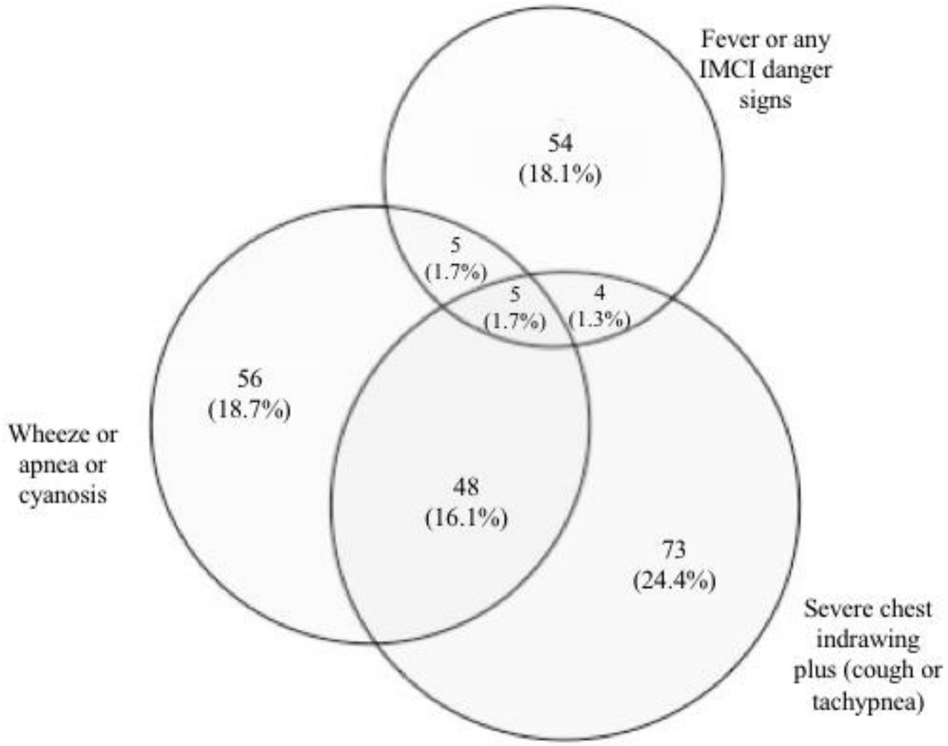

Supplement: Supplementary file 1 [file inf-38-e57-s001.pdf]
